# The Efficiency of Gene Electrotransfer in Breast-Cancer Cell Lines Cultured on a Novel Collagen-Free 3D Scaffold

**DOI:** 10.3390/cancers12041043

**Published:** 2020-04-23

**Authors:** Elisabetta Sieni, Monica Dettin, Mariangela De Robertis, Bianca Bazzolo, Maria Teresa Conconi, Annj Zamuner, Ramona Marino, Flavio Keller, Luca Giovanni Campana, Emanuela Signori

**Affiliations:** 1 Department of Theoretical and Applied Sciences, University of Insubria, 21100 Varese, Italy; 2 Department of Industrial Engineering, University of Padova, 35131 Padova, Italy; monica.dettin@unipd.it (M.D.); annj.zamuner@unipd.it (A.Z.); 3 CNR-Institute of Biomembrane, Bioenergetics and Molecular Biotechnology, 70126 Bari, Italy; m.derobertis@ibiom.cnr.it; 4 Department of Bioscience, Biotechnology and Biopharmaceutics, University of Bari, 70126 Bari, Itay; 5 Department of Pharmaceutical and Pharmacological Sciences, University of Padova, 35131 Padova, Italy; bianca.bazzolo@studenti.unipd.it (B.B.); mariateresa.conconi@unipd.it (M.T.C.); 6 Campus Bio-Medico University of Rome, 00128 Roma, Italy; r.marino@unicampus.it (R.M.); f.keller@unicampus.it (F.K.); 7 Department of Surgical Oncological and Gastroenterological Sciences DISCOG, University of Padova, 35124 Padova, Italy; luca.campana@unipd.it; 8 CNR-Institute of Translational Pharmacology, 00133 Roma, Italy

**Keywords:** Gene Electro-Transfer (GET), electroporation, 3D cell cultures, scaffold, breast cancer

## Abstract

Gene Electro-Transfer (GET) is a powerful method of DNA delivery with great potential for medical applications. Although GET has been extensively studied in vitro and in vivo, the optimal parameters remain controversial. 2D cell cultures have been widely used to investigate GET protocols, but have intrinsic limitations, whereas 3D cultures may represent a more reliable model thanks to the capacity of reproducing the tumor architecture. Here we applied two GET protocols, using a plate or linear electrode, on 3D-cultured HCC1954 and MDA-MB231 breast cancer cell lines grown on a novel collagen-free 3D scaffold and compared results with conventional 2D cultures. To evaluate the electrotransfer efficiency, we used the plasmid pEGFP-C3 encoding the enhanced green fluorescent protein (EGFP) reporter gene. The novel 3D scaffold promoted extracellular matrix deposition, which particularly influences cell behavior in both in vitro cell cultures and in vivo tumor tissue. While the transfection efficiency was similar in the 2D-cultures, we observed significant differences in the 3D-model. The transfection efficiency in the 3D vs 2D model was 44% versus 15% (*p* < 0.01) and 24% versus 17% (*p* < 0.01) in HCC1954 and MDA-MB231 cell cultures, respectively. These findings suggest that the novel 3D scaffold allows reproducing, at least partially, the peculiar morphology of the original tumor tissues, thus allowing us to detect meaningful differences between the two cell lines. Following GET with plate electrodes, cell viability was higher in 3D-cultured HCC1954 (66%) and MDA-MB231 (96%) cell lines compared to their 2D counterpart (53% and 63%, respectively, *p* < 0.001). Based on these results, we propose the novel 3D scaffold as a reliable support for the preparation of cell cultures in GET studies. It may increase the reliability of in vitro assays and allow the optimization of GET parameters of in vivo protocols.

## 1. Introduction

Gene Electro-Transfer (GET) is a versatile and efficient method that involves the use of Electroporation (EP) after the injection of nucleic acids (plasmid DNA, RNA, or oligonucleotides) into target tissues. This approach relies on the application of controlled electric pulses on a target tissue that induces the transient permeabilization of the cell membrane and the consequent uptake of therapeutic nucleotide sequences [1,2].

In the last two decades, the plasmid-based GET strategy has been successfully applied in preclinical models of neoplastic and other diseases, which was demonstrated to be a safe and effective approach [3,4,5,6,7]. Consequently, several clinical trials based on GET have been started [8] and initial promising results have been obtained in a first phase I clinical trial, where 10% of the patients with metastatic melanoma were successfully treated by transfecting a plasmid encoding interleukin-12 (IL-12) [9].

Although EP is a safe and efficient method for introducing genes into cells, many efforts are in place to optimize GET protocols to minimize tissue injury and enhance gene transfection efficiency by selecting the most appropriate electrical parameters [10,11,12]. It is well known, for instance, that EP parameters should be customized when applied to different tissues to produce the most effective electric field intensity [13,14].

Historically, cell suspensions and 2D cell cultures have represented the first in vitro models to investigate EP parameters [15,16,17,18,19]. However, in cell suspensions, the conductivity depends on the selected EP threshold [15,16]. More importantly, both approaches do not reliably mimic the in vivo tumor tissue architecture because of a lack of cell-extracellular matrix (ECM) connections or, in the case of 2D cultures, reciprocal cell–cell interactions typical of the 3D environment. Recently, tumor spheroids and hydrogels have emerged as suitable approaches to reproduce the complexity of tumor tissue architecture in EP-based studies to overcome these limitations [20,21,22,23,24,25,26,27,28].

These models are easy to manage but have intrinsic limitations. Spheroids lack reproducibility and show significant heterogeneity in size [29], which in turn significantly influences tumor sensitivity to electric fields [19,30,31,32], and spheroids simulate the in vivo environment only partially, due to the lack of cell–ECM interactions [20,31].

For all these reasons, recent studies have underlined the importance of reliable 3D tumor tissue models that may serve both to increase in vitro assay reliability and as a system to test accurately and modulate experimental conditions to propose for translational studies [33,34,35].

Scaffolds with different compositions have been investigated to design tumor-mimetic 3D platforms, such as type I collagen, hyaluronic acid, alginate, silk fibroin, and synthetic biocompatible polymers. Collagen is a key determinant of the tensile properties of breast connective tissue [36] and mammary tumors, which is characterized by increased collagen content and remodeling processes [37]. On the other hand, overproduction of other ECM components like fibronectin heparan sulfate, proteoglycans, and hyaluronic acid plays a significant role in breast cancer progression and metastasis [38]. In particular, hyaluronic acid is a unique nonsulfated glycosaminoglycan that is overexpressed in breast cancer and contributes to cancer cell growth and functional properties [39].

Both collagen and hyaluronic acid-based scaffolds have been already exploited as 3D ECM models to study tumor development, progression, and to test anticancer drugs [36,40,41]. However, from a chemical point of view, regarding type I collagen, it has been highlighted that the matrix microstructure depends on the origin of the product (rat tail, bovine skin, or human placenta) [42]. In contrast, hyaluronic acid is structurally invariant in the repetition of its dimer and is not subject to variation deriving from the source [42].

Regarding the use of 3D models for the definition of suitable EP-based in vitro protocols, many aspects should be considered. For instance, the electrical properties of tissue depend on different parameters, such as cell size and density, cell histotype, ECM structure (myxoid stroma, fibrous stroma, etc.), and the tissue characteristics that influence cell density and electric field distribution. As a result, different effects can be observed when EP is applied to different tissues. In the present study, we investigated GET efficiency by using HCC1954 and MDA-MB231 breast cancer cell lines grown on a novel collagen-free 3D scaffold. These cell lines were chosen as representative of primary and metastatic breast cancer, characterized by a different morphology associated with the stromal component, respectively. HCC1954 is a poorly differentiated cell line derived from a primary stage IIA, grade 3 invasive ductal carcinoma with no lymph node metastases and has slow stromal content [43]. In contrast, the MDA-MB231 cell line is derived from metastatic ductal breast carcinoma and presents abundant stroma with fibrous consistency [44].

Both HCC1954 and MDA-MB231 breast cancer cell lines were grown in a collagen-free scaffold based on crosslinked and lyophilized matrix components, such as hyaluronic acid and ionic-complementary self-assembling peptides (SAPs) condensed with the IKVAV (Ile-Lys-Val-Ala-Val) Laminin adhesion motif [45,46,47]. We previously demonstrated that it could reliably reproduce in vivo tissue architecture [48,49]. This scaffold result was particularly suitable to reproduce a tumor microenvironment where cells are embedded in a myxoid stroma, characterized by abundant amounts of hyaluronic acid and proteoglycans, low collagen, and elastin content [50].

In the present study, the collagen-free 3D scaffold was used, for the first time, to test GET protocols in a more realistic setting, similar to in vivo conditions.

## 2. Results

### 2.1. Morphology of HCC1954 and MDA-MB231 3D Cell Cultures

**Figure 1:**
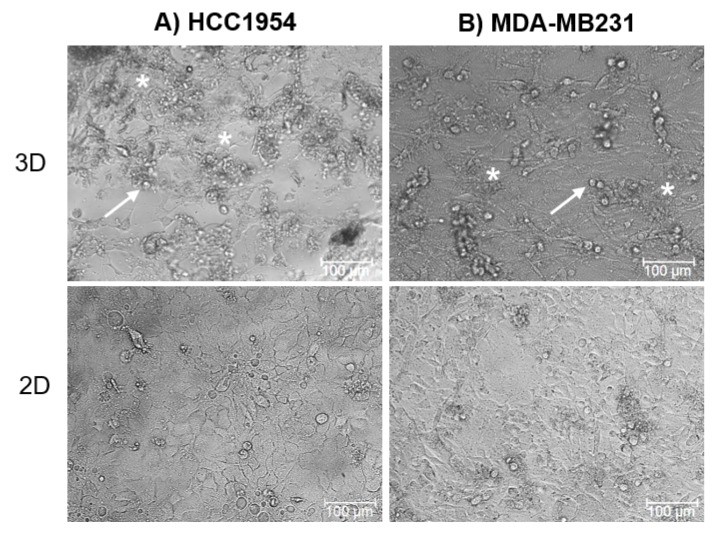
Cell morphology in 3D and 2D cultures. HCC1954 (**A**) and MDA-MB231 (**B**) 3D and 2D cell culture images acquired in a bright field. White arrows indicate cells, whereas white stars indicate the extracellular matrix deposed by cells. Data are from triplicate wells for each condition in two independent experiments. Scale bar 100 µm. Magnification 20×.

HCC1954 and MDA-MB231 cells cultured on the 3D scaffold compared with their classical 2D cell culture (Figure 1) showed peculiarities in cell morphology and ECM deposition.

**Figure 2:**
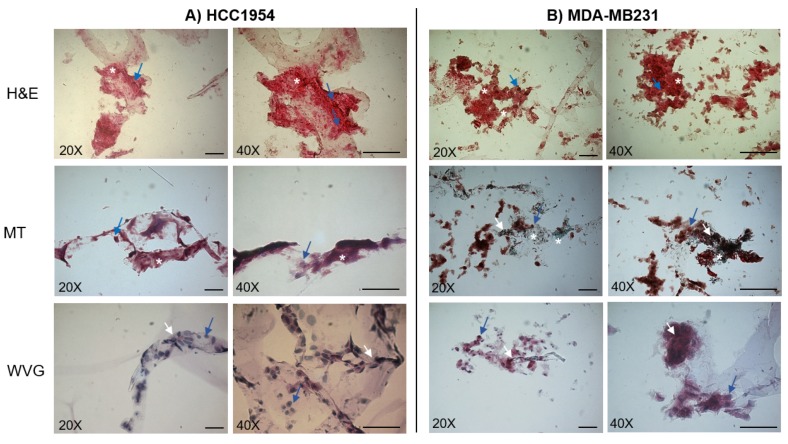
Histological images of HCC1954 (**A**) and MDA-MB231 (**B**) cells cultured in the 3D scaffold for seven days. Representative images acquired by an inverted microscope after staining with Hematoxylin and Eosin (H&E), Masson Trichrome (MT), and Wiegert Van Gieson (WVG) methods. The blue arrows indicate cells, the white stars the extracellular matrix, and the white arrows indicate collagen. Data are from triplicate wells for each condition in two independent experiments. Scale bar 100 µm. Magnification 20× and 40×.

All cells cultured on the 3D scaffold were more round or polygonal-shaped compared to 2D cultures, where the attachment to the plastic plate bottom induced a visible spreading of cells. In particular, in the MDA-MB231 3D culture, the cells were round-shaped with an average diameter of around 18 µm. A few tens of cells were revealed in an area of 800 × 500 µm. On the other hand, HCC1954 cells grown in the 3D scaffold had an average diameter of around 15 µm and we found about 15 cells in an area 150 × 150 µm (Figure 1 and Figure 2). Moreover, cells in the 3D scaffold produced ECM, as indicated in Figure 1.

The histological examination of both the MDA-MB231 and HCC1954 3D cell cultures (Figure 2) performed by the Hematoxylin and Eosin (H&E), Masson Trichrome (MT), and Wiegert Van Gieson (WVG) staining methods after seven days of incubation in the new scaffold revealed that cells made a lot of cell–cell connections and were attached to the scaffold and the new deposed ECM. In some regions, the cells (blue arrows) appear faintly visible respect to the surrounding ECM components (white stars).

As shown in Figure 2, HCC1954 and MDA-MB231 cells in 3D cell culture showed different spatial organization, bounding to the scaffold and production of ECM components. In particular, HCC1954 cells in 3D cell culture were round-shaped, disjointed, uniformly attached to the scaffold structure, and surrounded by copious ECM. Inversely, the MDA-MB231 cells cultured in the 3D scaffold were strictly joined to each other and produced a denser ECM, which appeared more abundant in some areas, thus creating an inhomogeneous distribution of cell clusters and dense ECM areas.

In both HCC1954 and MDA-MB231 3D cell cultures, the ECM composition was investigated. Samples staining with the WVG and the MT methods highlighted the production of collagen (green or red color) and other connective tissue components (white arrow). The WVG staining of HCC1954 cells revealed abundant collagenous fibers and poorly represented components of connective tissue, whereas, in the MDA-MB231 3D cell culture, the connective stroma was visible (MT staining). MT staining showed that in both 3D cell cultures, there was substantial collagen deposition. This indicates the ability of the new 3D scaffold to allow the establishment of a tumor microenvironment enriched in matrix protein polymers, such as the collagens, as widely reported in different tumor tissues [51].

### 2.2. GET Protocol Efficiency in 3D and 2D Cell Cultures Using Linear Electrodes

**Figure 3:**
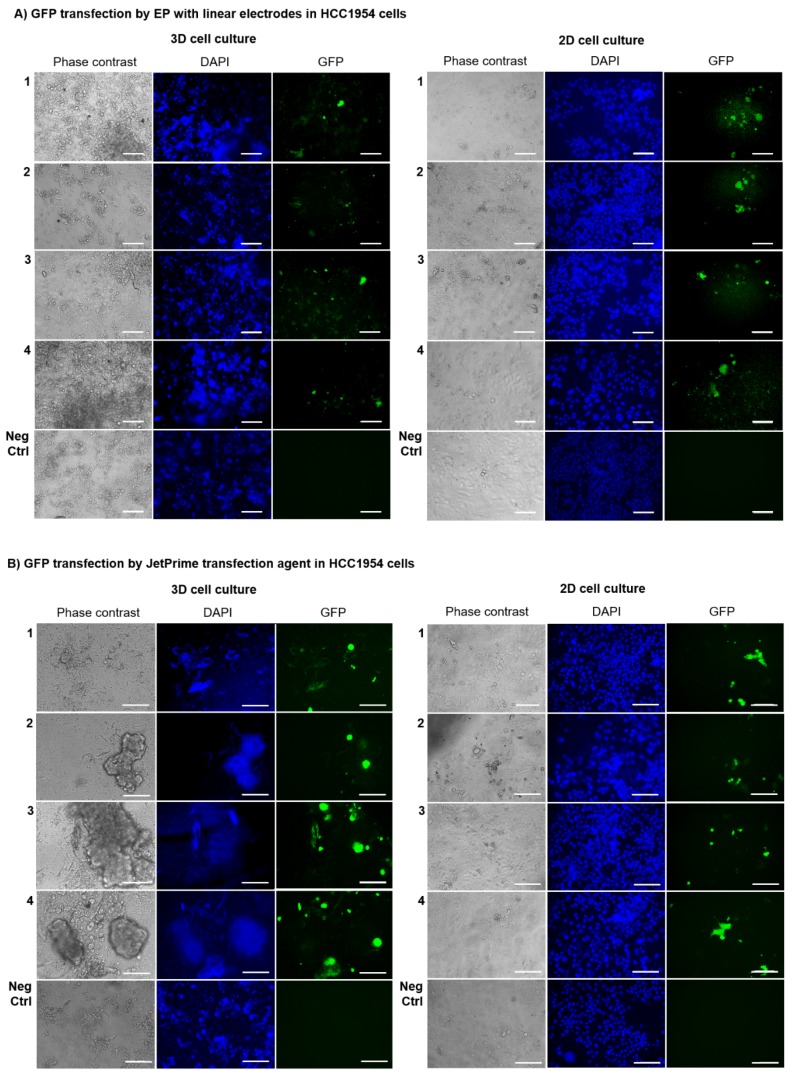

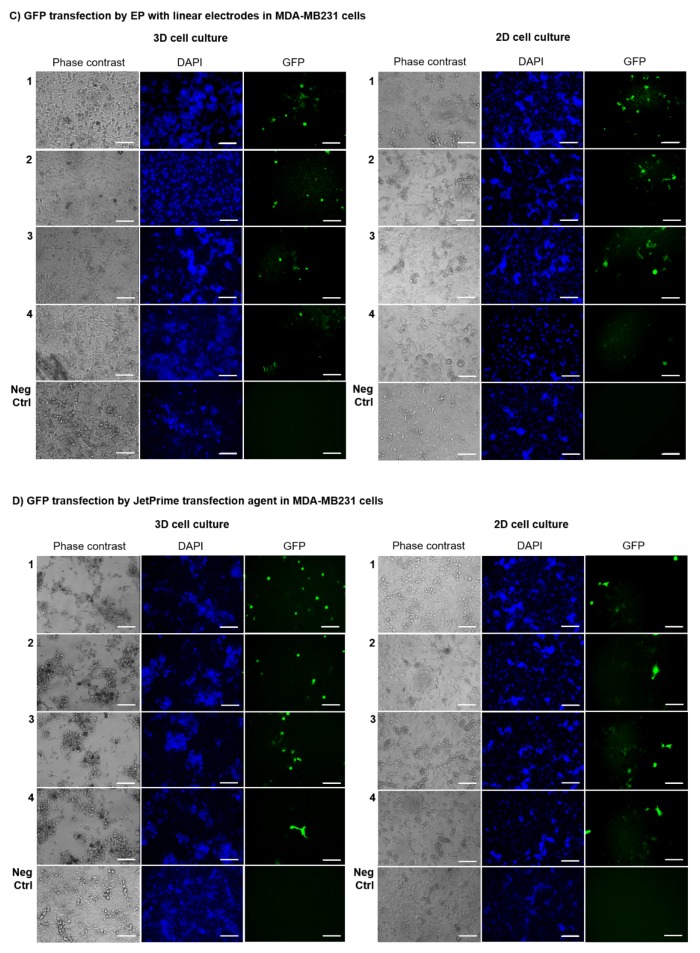
Efficiency of different Gene Electro-Transfer (GET) protocols in HCC1954 and MDA-MB231 cells according to the modality of cell culture (3D versus 2D). Cells were transfected with Electroporation (EP) employing linear electrodes or JetPrime^®^ transfection agent. Representative fluorescence images of HCC1954 (**A,B**) and MDA-MB231 (**C,D**) 3D and 2D cell culture transfected with green fluorescent protein (GFP) by EP with linear electrodes (**A,C**) and by JetPrime^®^ transfection agent (**B,D**). Representative micrographs of cells in bright field and using 4′,6-Diamidino-2-Phenylindole (DAPI) and GFP filters. Micrographs are representative of two separate blind experiments with similar outcomes. Not transfected cells are the Negative Control (Neg Ctrl). Observation at three days post-transfection. Magnification 20×. Scale bars, 100 µm.

HCC1954 and MDA-MB231 cell lines were grown in the new collagen-free 3D scaffold and underwent the application of a GET protocol consisting of EP-based transfection of the plasmid pEGFP-C3 using linear electrodes (8 voltage pulses, 1300 V/cm, 100 μs long at 1 Hz). These electrical parameters were previously demonstrated to assure a high EP efficiency in cells seeded in monolayer or the same 3D scaffold [50]. To verify the reliability of the novel 3D scaffold as an ex vivo model for GET protocols, we applied EP through linear electrodes on HCC1954 and MDA-MB231 3D cell cultures. The results were compared to the corresponding 2D cell cultures as control (Figure 3A,C; Supplementary Table S1).

HCC1954 and MDA-MB231 3D and 2Dcell cultures were treated in parallel using the JetPrime^®^ transfection agent (Figure 3B,D) and were considered as the positive control, due to the reported JetPrime^®^ good efficiency of transfection on a wide variety of cell lines, also resulting in very low cytotoxicity. We were able to evaluate the GET protocols efficiency on the cells seeded in the new scaffold or monolayer by considering the percentage of green fluorescent protein (GFP)-positive cells compared to the total cell number on both 3D versus 2D breast cancer cell cultures after three days post GET or JetPrime^®^-mediated transfection. Data were obtained from three replicates per experimental condition; all the experiments were repeated twice in a blind fashion.

**Figure 4:**
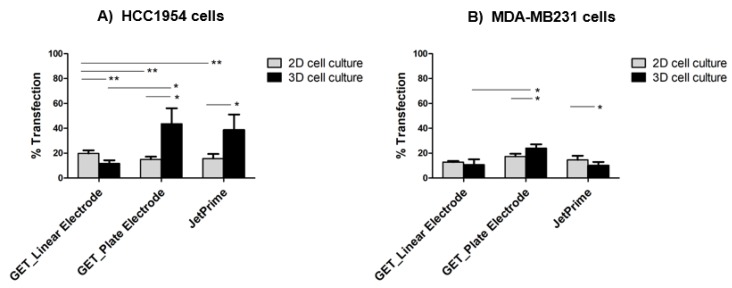
Percentage of cell transfection of HCC1954 (**A**) and MDA-MB231 (**B**) cell lines in 2D and 3D cultures measured three days after EP (with linear or plate electrode) or JetPrime^®^. Data are from three replicates per experimental condition, in two independent blind experiments (Mean ± SD). Statistical analysis was performed using the Student’s *t*-test (* *p* < 0.01, ** *p* < 0.001, *** *p* < 0.0001).

Figure 3 shows that the overall transfection efficiency, in terms of percentage of transfection, was more homogeneous in 2D-cultured HCC1954 and MDA-MB231 cells (15%–20% of transfection efficiency using either GET or JetPrime^®^) than HCC1954 and MDA-MB231 3D-cultured cells where, probably, the presence of the scaffold emphasized the difference between the two cell lines, reflecting the peculiar tumor histology of the tissues of origin. Besides, transfection efficiency was slightly lower in HCC1954 3D cell culture than in HCC1954 2D cell culture treated with the GET protocol using the linear electrode (12% in HCC1954 3D cell culture versus 20% in HCC1954 2D cell culture, *p* < 0.001) (Figure 3B and Figure 4A).

Moreover, no significant differences were observed between MDA-MB231 cells cultured in 2D or 3D system and treated with the GET protocol using linear electrodes (13% in MDA-MB231 3D cell culture versus 11% in MDA-MB231 2D cell culture, *p* not significant) (Figure 3C and Figure 4B). As positive control, the application of JetPrime^®^ transfection to either HCC1954 or MDA-MB231 cells grown in 3D or 2D cultures gave a transfection efficiency of 16% in HCC1954 3D cell culture versus 19% in HCC1954 2D cell culture, *p* < 0.01, and 14% in MDA-MB231 3D cell culture versus 17% in MDA-MB231 2D cell culture, *p* < 0.01).

Overall, the percentage of transfection efficiency was higher in all the experiments performed in 3D HCC1954 cells than in 3D MDA-MB231 cells, showing not only a different propensity of these breast cancer cell lines to uptake plasmid DNA by GET-based transfection protocols but also the ability of the 3D cultures to maintain and emphasize the cell line difference.

### 2.3. GET Protocols Efficiency in 3D Cell Cultures Using Plate Electrodes

**Figure 5:**
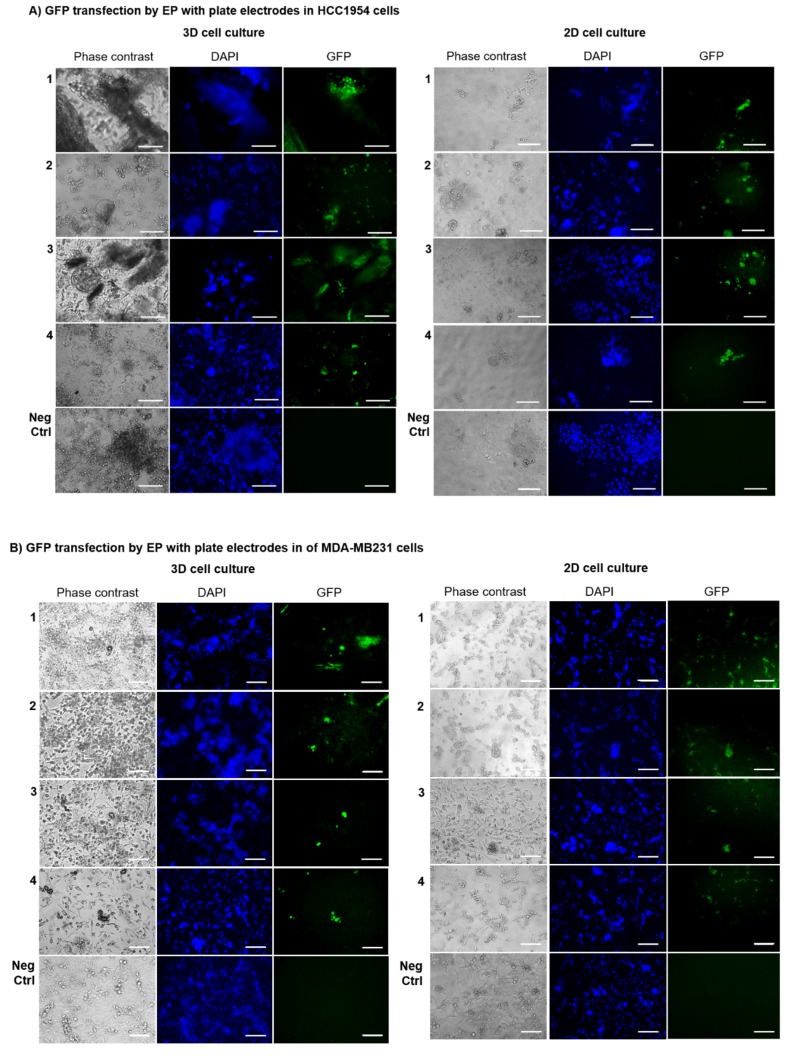
The efficiency of different GET protocols in HCC1954 and MDA-MB231 3D and 2D cell cultures transfected by EP with plate electrodes. Representative fluorescence images of (**A**) HCC1954 and (**B**) MDA-MB231 3D and 2D cell cultures transfected with GFP by EP with plate electrodes. Observation at three days post-transfection. Representative micrographs of cells in bright field and using DAPI and GFP filters. Micrographs are representative of two separate blind experiments with similar outcomes. Not transfected cells are the Negative Control (Neg Ctrl). Magnification 20×. Scale bars, 100 µm.

A different GET protocol, based on the intratumoral transfection of the plasmid pEGFP-C3 through the EP approach utilizing plate electrodes, was evaluated in the HCC1954 and MDA-MB231 cells grown in the collagen-free 3D scaffold. The same EP parameters previously described for linear electrodes were applied (8 voltage pulses, 1300 V/cm, 100 μs long at 1 Hz). Figure 5 illustrates the effect of the application of plate electrodes for GET-based GFP transfection on the number of transfected cells, showing better results in 3D-cultured cells compared to the respective 2D-cultured ones (Supplementary Table S2).

A more evident increase of the overall transfection efficiency was observed in HCC1954 3D cell culture (44%) compared both to the respective 2D cell culture (15%, *p* < 0.01) and to the HCC1954 3D cell culture treated with linear electrodes (12%), *p* < 0.001 (Figure 4A and Figure 5A). A similar trend was found in MDA-MB231 cells cultured on the 3D scaffold and transfected with plate electrodes, which showed a transfection efficiency of 24% compared to 17% in 2D culture, *p* < 0.01. Moreover, the transfection efficiency was significantly higher compared to the results obtained in the MDA-MB231 cells in 3D culture treated with linear electrodes (11%), *p* < 0.01 (Figure 5A,B).

### 2.4. HCC1954 and MDA-MB231 3D Cell Culture Viability after GET Using Plate vs Linear Electrodes

**Figure 6:**
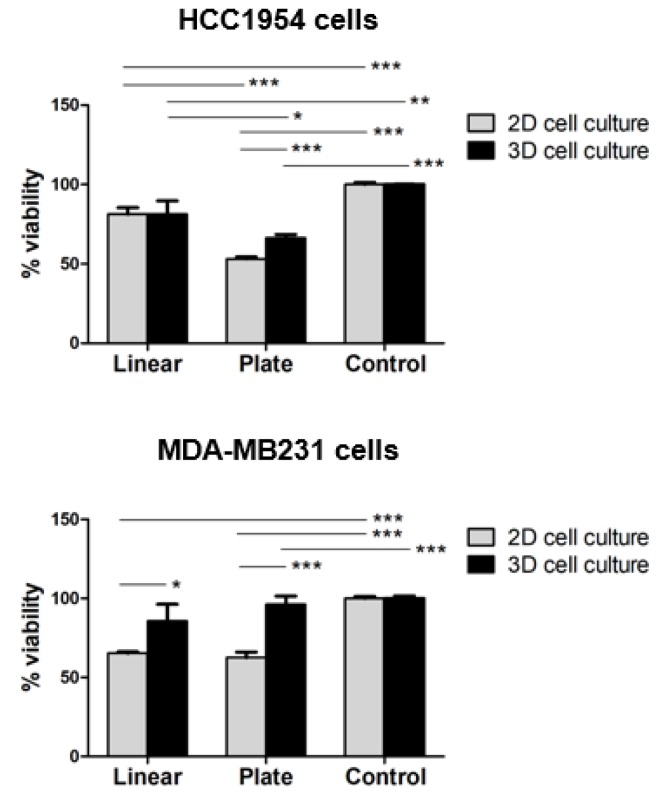
Viability of HCC1954 and MDA-MB231 2D and 3D cell culture after three days post-GET using linear or plate electrodes. Percentage (%) viability obtained using PrestoBlue^TM^ Cell Viability assay. Control: untreated cells. Graphs represent quadruplicate biological repeats analyzed blindly and are displayed as Mean ± SD. Statistical analysis was performed using the Student’s *t*-test. * *p* < 0.01, ** *p* < 0.001, *** *p* < 0.0001.

Since EP conditions (pulsing parameters, electrode shapes) and characteristics of the cell culture (2D versus 3D in vitro models) directly influence membrane permeabilization and consequently the cell viability, we evaluated the viability of HCC1954 and MDA-MB231 cells cultured as a monolayer and in the new 3D scaffold at three days post-GET using linear vs plate electrodes. We did not assess cell viability after JetPrime^®^ transfection because according to the manufacturer’s instructions it is known that JetPrime^®^ reagent is very gentle on cells since it requires low amounts of reagent and nucleic acid during transfection. The PrestoBlue^TM^ Cell Viability assay (Figure 6) revealed, in most cases, a reduction of cell survival for both HCC1954 and MDA-MB231 cells cultured in the 3D and 2D compared to the respective controls.

In particular, the viability of both HCC1954 and MDA-MB231 cells cultured in the 3D scaffold for three days after GET, performed using either plate (66% and 96%, respectively) or linear (81% and 86%, respectively) electrodes, was higher than viability in the correspondent 2D cell cultures using either plate (53% and 63%, respectively) or linear (81% and 65%, respectively) electrodes (*p* < 0.001 for the plate electrode; *p* < 0.01 for the linear electrode in MDA-MB231 cells). HCC1954 cells did not exhibit any difference between 3D and 2D cells viability. Moreover, the use of the linear electrode in HCC1954 cells was significantly associated with lower cell mortality in both 2D and 3D cell culture (*p* < 0.001 for 2D cell culture; *p* < 0.01 for 3D cell culture). In contrast, there were no significant differences in the viability of MDA-MB231 cells subjected to GET using either linear or plate electrodes.

## 3. Discussion

In this work, we used a new collagen-free 3D scaffold to test GET-based protocols efficiency in breast cancer HCC1954 and MDA-MB231 3D cell cultures, and it proved to be a promising model for the investigation of intratumoral plasmid-based gene delivery strategies.

This type of collagen-free 3D scaffold is based on crosslinked and lyophilized matrix components, such as hyaluronic acid and SAPs carrying the IKVAV adhesion sequence. IKVAV, a small peptide derived from laminin-111, promotes not only cell adhesion but also induces tumor growth, metastasis, activation/secretion of proteases, and angiogenesis [52].

Compared to different collagen-based 3D scaffolds, our hyaluronic acid-based matrix showed several useful features. First, the molecules constituting the 3D scaffold represent a protein component with specific sequences for cell adhesion. Moreover, as obtained by chemical synthesis, they ensure, unlike Matrigel, a precise composition that is invariant from batch to batch [53]. Further, this new 3D scaffold can successfully reproduce some ECM features of a ‘pre-cancerous state’ where cells are surrounded by a myxoid stroma, mainly composed by abundant basic substances with large amounts of glycosaminoglycans (hyaluronic acid) and proteoglycans, poor collagen fibers (SAPs) and no elastin content. Notably, as a third important feature of this novel 3D scaffold, our study confirmed that in this type of scaffold it is possible to appreciate the matrix remodeling and de novo deposition of ECM rich in collagen by cancer cells growing in 3D cell culture as that observed in human mammary tumors.

We previously reported on EP experiments on breast cancer cells seeded in such a collagen-free 3D scaffold [50]. Thanks to the presence of cell–cell and cell–ECM interactions, the new 3D scaffold may represent more reliable support for EP studies than 2D cancer cell cultures, and it may be used to test new EP-delivered drugs and novel EP protocols [50].

Here, to test the new 3D scaffold for the study of novel GET-based protocols, we used two breast cancer cell lines with different histological features. In particular, the HCC1954 cell line was derived from a primary ductal carcinoma without lymph node metastases and a low stromal content [43], while the MDA-MB231 cell line originated from a metastatic ductal carcinoma, characterized by abundant tissue stroma [44]. We evaluated HCC1954 and MDA-MB231 cells cultured as a monolayer as well as in the novel 3D scaffold and compared cell organization, the efficiency of different GET protocols and cell viability as a function of different gene transfection conditions.

Concerning cell organization, our results showed that cells cultured for seven days in the 3D scaffold (3D cell culture) or as monolayers (2D cell culture) had a distinct morphology and spatial disposition, following the main features of the respective tumor of origin. As shown in Figure 1, all cells in the 3D scaffold were more round- or polygonal-shaped than the respective 2D cultured ones, due to the adhesion to the plastic plate bottom, with a visible dispersion of cells. This observation was in line with previous findings indicating that the composition and the biomechanical properties of a 3D scaffold can influence the morphological phenotype of cancer cells [54]. For instance, it was previously reported that HCC1954 cells in 2D culture are significantly spread out and do not form groups or colonies, while using the forced-floating poly-HEMA method of 3D culture HCC1954 cells form tight spheroids, with a slightly smooth surface [55]. MDA-MD231 3D cell culture organization in Matrigel is classified as ‘stellate’ with a not-well definite shape that in some cases could be approximated as round and in others as elongated and with invasive processes [54].

In our study, we confirmed such distinct 3D morphology between HCC1954 and MDA-MB231 cells, but HCC1954 cells in our 3D scaffold of hyaluronic acid and SAPs grew generally separated, uniformly attached to the scaffold structure, and surrounded by copious ECM (Figure 1 and Figure 2). Conversely, the MDA-MB231 cells cultured in the 3D scaffold were strictly joined to each other and produced an inhomogeneous distribution of cell clusters and thicker ECM areas (Figure 1 and Figure 2). When considering Figure 1 and Figure 2**,** it is also evident that in the new 3D scaffold, both HCC1954 and MDA-MB231 cells were able not only to form small aggregates but also to produce ECM.

The evaluation of the ECM composition by staining with the WVG and the MT methods in both the 3D cell cultures revealed that thanks to the 3D scaffold, HCC1954 and MDA-MB231 cells produce connective tissue components and substantial collagenous fibers deposition (Figure 2). Our results showed that the 3D-cultured MDA-MB 231 cells were surrounded by a fibrous stroma, marked with white crosses in Figure 2, with some elongated or rounded cells, arranged in groups, attached to the scaffold structure (blue arrows in Figure 2). Concerning the HCC1954 cell line, the cells in the 3D scaffold were attached to the scaffold and surrounded by the stroma that was composed in prevalence by collagen fibers, as revealed by MT and WVG staining (white arrows in Figure 2).

Notably, as a piece of first important evidence, we observed that the biomechanical characteristics of the new 3D scaffold and the nature of the tumor cells jointly influence the architecture of the in vitro tissue model. Moreover, the 3D scaffold of hyaluronic acid and SAPs induces a consistent production and deposit of ECM components around cells, thus reproducing a common feature of several human breast malignancies that show an abundant fibrous component, with collagen constituent, and scarce cellular component [38,56,57]. We showed that, as further demonstrated in [50], our 3D model can represent a suitable experimental platform to test GET protocols on mammary tumor cells surrounded by ECM.

Based on the results of the histological validation, the new 3D model was used to evaluate the transfection efficiency of intratumoral plasmid DNA-based gene transfer in more realistic conditions. The conditions, closer to those observed in pathological tissues, are characterized by the ECM presence and the tissue inhomogeneity ascribable to fibrous component and local cell density differences. To verify the reliability of the novel 3D scaffold when used in different GET protocols, and to identify the best GET conditions, two different transfection methods—EP with linear or plate electrodes (Figure 3 and Figure 5) or JetPrime^®^ transfection agent (Figure 3B,D)—were tested on 3D cell cultures, using the respective HCC1954 and MDA-MB231 2D cell culture as controls. Considering that the topology and the size of the plasmid DNA vector influence the efficiency of transfection, we focused our analysis on the transfection with a single plasmid construct. In particular, we used the plasmid pEGFP-C3 (4.7 kb), which is a common vector frequently used in model systems of DNA transfection [58]. The voltage pulse parameters used in our study, consisting of applying eight voltage pulses (1300 V/cm, 100 μs long at 1 Hz), were previously demonstrated to assure a high EP efficiency in cells cultured in the same 3D scaffold [50]. The evaluation of the percentage of cells positively transfected with GFP after three days post GET or JetPrime^®^-mediated transfection of the plasmid pEGFP-C3, revealed mixed results, in terms of transfection efficiency, in both HCC1954 and MDA-MB231 3D-cultured cells compared to the respective 2D-cultured ones, which lacked the realistic complexity of the respective 3D models. It is well known that tissue inhomogeneity could influence the electric field distribution [59,60,61,62]. Therefore, the variable results we obtained in the 3D cell cultures could be attributed to the presence of the scaffold, which was likely able to emphasize the intrinsic differences between HCC1954 and MDA-MB231 cells. In particular, the HCC1954 is a poorly differentiated cell line derived from a primary grade 3 invasive ductal carcinoma with no lymph node metastases [44]. In contrast, the MDA-MB231 cell line commonly used to model late-stage breast cancer was derived from an infiltrating ductal breast carcinoma, where the fibrous stroma is well visible with a loose or dense consistency [45].

Next, our results revealed a lower transfection efficiency in HCC1954 3D cell cultures than in the respective 2D cell cultures using either the GET protocol (*p* < 0.001) (Figure 3A and Figure 4A) or the JetPrime^®^ transfection (*p* < 0.01) (Figure 3B and Figure 4A). Although we observed a higher percentage of transfection efficiency in the experiments performed in HCC1954 cells, consistent results were also obtained in MDA-MB231 cells. These results show lower transfection efficiency in cells cultured in the 3D scaffold compared to the respective 2D culture, using GET with linear electrodes (*p* < 0.01) (Figure 3D and Figure 4B). This could be due not only to a particular 3D disposition of cells in the scaffold, including several cell–cell and cell–matrix interactions but also to the effect of a significant amount of ECM.

Furthermore, we observed a higher transfection efficiency following the application of plate electrodes in both HCC1954 and MDA-MB231 3D-cultured cells compared to the respective 2D culture (*p* < 0.0001 and *p* < 0.01, respectively) (Figure 5). This finding suggests a better performance of our GET conditions using plate electrodes. This result was in line with in vivo models, experimentally validated, showing that the diameter of the needles used to apply electric pulses influences the field distribution in tissues: the thinner the needles, the less homogeneous the electric field distribution [63]. Moreover, the different GET efficiency obtained by using a plate or linear electrodes could be ascribed to the gaps between electrodes, with the plates being 7 mm apart and the linear being 4 mm apart. Therefore, it is possible that from one side more cells were exposed to the electric field and from the other side, a higher field was applied with the plate electrode than the linear one.

Finally, since developing a reliable ex vivo model for testing GET-based protocols with high efficiency and viability is critical for clinical application, but the exposure of cells to high electric fields strengths can lead to cell death due to irreversible EP or due to EP-induced indirect effects, we investigated also cell viability.

Interestingly, through the PrestoBlue^TM^ Cell Viability assay, we observed that viability of both HCC1954 and MDA-MB231 cells cultured in the 3D scaffold for three days after GET performed using plate or linear electrodes was higher than viability in 2D cell culture, using either the plate electrode (*p* < 0.001) or the linear one (*p* < 0.01) (Figure 6). HCC1954 cells treated with the linear electrode did not exhibit any difference between 3D and 2D cell viability. Moreover, no significant difference was observed between the use of linear or plate electrodes in MDA-MB231 cells considering together 2D and 3D cell culture, while HCC1954 2D and 3D cell cultures were significantly associated with a lower cell mortality (*p* < 0.001 for 2D cell culture; *p* < 0.01 for 3D cell culture, Figure 6). This may suggest that the presence of the 3D scaffold might preserve the cell culture from the EP-induced effects on cell mortality. These data indicate that the 3D scaffold might exert a protective role against the EP-dependent mortality of cells. According to a recent study on Irreversible Electroporation (IRE) performed on 3D-cultured cancer cells in dense collagen I hydrogels [24], we suppose that because of cell protrusions interacting with the collagen matrix, the cell diameters in the 3D model are larger than they are in 2D suspension. This may contribute to increasing the electric-field threshold for cell death, being a function of the cell diameter increment [64]. This makes the proposed 3D model more suitable to test the GET conditions to translate in vivo, also encouraging to test more stringent conditions, such as the application of higher electric field intensity, compared to the parameters evaluable in 2D cell cultures.

Taken together, our results show that the new collagen-free 3D scaffold is a reliable model for in vitro studies on EP-based gene drug delivery.

Concerning this aspect, despite a certain level of efficacy of intratumoral delivery of plasmid vectors in human studies, it would be ideal to gauge the electrical parameters accordingly to the features of each tumor type, particularly in those lesions where tissue composition may impede the propagation of electric currents so affecting gene delivery [65]. As we previously reported using different EP protocols [50], conductivity settings in the 3D model are very similar to those of the stroma tissue. Importantly, our model is reliable in terms of EP transfection efficiency and cells viability, thus allowing to reduce the number of preclinical studies in animal models—with more favorable ethical and economic implications in this field—and to lead to a possible acceleration of the time gap between preclinical and clinical studies.

## 4. Materials and Methods

### 4.1. 3D Scaffold Preparation

The self-assembling peptide (SAP) functionalized with Laminin adhesion sequence was synthesized by Fmoc chemistry using Rink Amide MBHA resin (0.7 mmol/g; scale 0.125 mmol) and the synthesizer Syro I (Multisynthec, Witten, Germany). The Rink Amide MBHA resin and the Fmoc protected amino acids were purchased from Novabiochem (Merck KGaA, Darmstadt, Germany).

SAP was an analog of the complementary ionic peptide of module II (called EAK) [66,67,68] with a substitution Ala→Abu (Abu = α-aminobutyric acid) and the addition of an IKVAV Laminin sequence at its C-terminal. The first three amino acids and the last sixteen amino acids were introduced through double couplings. The coupling reagents 2-(1H-Benzotriazole-1-yl)-1,1,3,3-tetramethyluronium hexafluorophosphate (HBTU) and 1-Hydroxybenzotriazole (HOBt) from Advanced Biotech (Seveso, MI, Italy). At the end of the synthesis, the Fmoc was removed, the resin was washed with dichloromethane (DCM) (Biosolve, Leenderweg, Valkenswaard, The Netherlands) and dried for 1 h under vacuum. The resulting peptide was removed from the solid support with contemporary side-chain deprotection using the following mixture: 0.125 mL MilliQ water, 0.125 mL Triethoxysilane (TES) (Sigma Aldrich, Steinheim, Germany), and 4750 mL trifluoroacetic acid (TFA) (Biosolve, Valkenswaard, The Netherlands) over 90 min, under magnetic stirring. Finally, the resin was filtered, and the reaction mixture was concentrated. Then, the crude peptide was precipitated using cold diethyl ether. The peptide was purified by reverse phase chromatography (RP-HPLC), characterization by analytical chromatography and its identity was ascertained by MALDI-TOF mass spectrometry (theoretical value = 2239 Da; experimental value = 2236.32 Da). The hyaluronic acid/SAP solution was obtained by adding 3% w/v Hyaluronic acid (MW = 100–1250 kDa, Contipro Biotech s.r.o, Dolni Dobrouc, Czech Republic) to 0.12% w/v SAP dissolved in MilliQ water. The resulting solution was divided into 5 wells of a chamber slide, frozen in liquid nitrogen and lyophilized. The scaffolds (dimension: 8 × 10 × 5 mm) were cross-linked through reaction with 50 mM 1-Ethyl-3-(3-dimethylaminopropyl)carbodiimide (EDC) (Sigma Aldrich, Steinheim, Germany) in 95% ethanol for 24 h. The scaffold was washed in an ultrasound bath twice with ethanol for 30 s and twice with MilliQ water for 30 s. Finally, the scaffold was frozen at −20 °C and lyophilized.

### 4.2. 2D and 3D Cell Cultures

Two breast cancer cell lines (HCC1954 and MDA-MB231) (ATTC, www.atcc.org) were cultured in RPMI and Dulbecco’s Modified Eagle Medium (DMEM) (Corning, Mediatech Inc., NY, USA), respectively. Both media were complemented with 100 U/mL Penicillin/Streptomycin (Gibco, Invitrogen, Carlsbad, CA, USA), 2 mM L-Glutamine (Sigma-Aldrich, St. Louis, MO, USA), and 10% fetal bovine serum (FBS, Gibco, Invitrogen, Carlsbad, CA, USA). Then, cells were seeded in parallel in 24-wells plates (5 × 10^4^/well) and inside the 3D scaffold (3 × 10^5^/cm^2^ in 500 μL of medium) previously hydrated for 1 hour in an ultra-low attachment 24-wells plate or chamber slide support. Both cells in the 24-wells plates (2D culture) and the scaffold (3D culture) were incubated at 37 °C, 5% CO_2_ concentration, and 95% relative humidity. The histological analysis of 3D cell cultures was performed after seven days of incubation. Both 2D and 3D cell cultures were treated with GET-based protocols after three days of culture in the chamber slide, then incubated and analyzed after three days. All experiments were performed in triplicate wells for each condition and repeated twice.

### 4.3. Histological Examination of 3D Cell Cultures

The histological analysis of the 3D cell cultures was performed after seven days of incubation. The 3D cell cultures were included in 2% agarose gel (Seakem^®^ LE agarose, Lonza, Rockland, ME, USA) and frozen at −20 °C. Cryo-sections (20 µm thickness) from frozen blocks were properly stained using three different staining techniques. First, the Hematoxylin and Eosin (H&E) stain (Bio-Optica, Milan, Italy) was used to evaluate cells and ECM features. Then, the Masson’s trichrome (MT) and Weigert Van Gieson (WVG) (Bio-Optica, Milan, Italy) were used as specific stains for collagen and connective tissues. In particular, the MT stains the ECM in red and the cells in a red-brown color, while the WVG dies in blue or in green the collagen fibers, in dark the cells and in red other extracellular matrix components. The stained slides were observed under a Leica DMR optical microscope (Leitz, Wetzlar, Germany) at 20x magnification, and the image data were acquired with a digital Nikon DSU-1 camera (Amsterdam, The Netherlands).

### 4.4. GET Protocols on 2D vs 3D Cell Cultures

To perform GET-based experiments both 2D and 3D cell cultures were cultured for three days in an 8-well chamber slide, in 500 and 700 uL of complete culture medium, respectively.

The EP protocol for both 2D and 3D cell cultures was performed using two different types of electrodes (Supplementary Figure S1), i.e., a plate and a linear electrode, and applying 8 voltage pulses (1300 V/cm, 100 μs long at 1 Hz), as reported in [39]. The plate electrode was formed by two 3 cm long, 1 cm large stainless-steel plates, with a gap of 7 mm, while the linear electrode was formed by a set of 8 needles organized in 2 parallel lines at 4 mm. The voltage pulses were applied by a pulse generator EPS-01 (Igea, Carpi (MO), Italy).

An amount of 250 ng and 5 µg of plasmid pEGFP-C3 was added in both 2D and 3D cultures, proportionally to cell density in each cell culture, as reported in [69]. Then the electrodes were immersed in the chamber slide to the well bottom. Finally, cells were incubated three days at 37 °C, 5% CO_2_ concentration, and 95% relative humidity. Each GET experiment was performed in triplicate wells for each condition and repeated twice in a blind fashion.

In parallel, both 2D and 3D cell cultures were transfected with pGFP-C3 using JetPrime^®^ (Polyplus-transfection S.A., Illkirch, France) according to the manufacturer’s instructions and were considered as the positive control.

### 4.5. Analysis of Cells Viability

Cell viability on the electroporated samples was assessed three days after GET by using the PrestoBlue™ Cell Viability Reagent (Thermo Fisher Scientific, Waltham, MA, USA) according to the manufacturer’s instruction. Briefly, at each time point, 10% of PrestoBlue™ was added directly to each well and incubated for 1 h at 37 °C. Fluorescence (excitation 560 nm, emission 590 nm) was read using the Victor plate reader (Victor3 1420 Multilabel Counter, PerkinElmer, Boston, MA, USA). Control samples were untreated cells.

All the cell viability experiments started with the same cell numbers (7.5 × 10^4^ cells in 2D and 1.8 × 10^5^ cells in 3D) and were performed as quadruplicate biological repeats in a blind fashion. Both 3D and 2D cell cultures were analyzed following the same time of culture after treatment with GET.

### 4.6. Fluorescence Microscopy

Three days after GET-based transfection of GFP, both 3D and 2D cell cultures were added with 5 μL of Hoescth 33342 fluorescent dye (1 mg/mL in PBS) and observed at inverted fluorescence microscope (Leica DMI4000, Leica Microsystems GmbH, Wetzlar, Germany) at a magnification of 20×. Bright-field images and fluorescence images using DAPI and GFP (DAPI excitation 361 nm, emission 486 nm; GFP excitation 488 nm, emission 510 nm) filter sets were acquired. All the images were acquired separately with a camera (ANDOR, Neo sCMOS, Nikon, Amsterdam, The Netherlands) and analyzed using Leica LAS AF Lite software. From the images, the number of transfected cells was determined as described in the following section.

### 4.7. Statistical Analysis

The number of GFP-positive cells was counted and converted to a percentage of the total cell number (100%) in three areas selected at random from the examined 2D and 3D cell cultures transfected with GFP using the plate or the linear electrode or the JetPrime^®^ transfection agent. The statistical significance of differences between sample groups, both for the quantitative evaluation of cells transfected with GFP and for the analysis of the cell viability assay, was assessed using unpaired Student’s *t*-test. Statistical analysis was performed using Prism 5 statistical software (GraphPad Software Inc., San Diego, CA, USA). All values are expressed as mean ± Standard deviation (SD). A *p*-value < 0.01 was considered statistically significant.

## 5. Conclusions

Over the last two decades, the progress of both in vitro and in vivo EP, coupled with the improvement of technological aspects, has contributed to bringing GET to the clinical stage.

The results of the first human trial with GET in patients with metastatic melanoma have been published [8] and indicate that this approach is feasible, safe, and associated with promising results.

Nonetheless, further progress is needed before a broader application. A first important step concerns the optimization of the GET protocols through more reliable in vitro and preclinical models.

We propose a new collagen-free and easy-to-prepare scaffold for 3D cell culture as a suitable platform for the optimization of EP parameters in GET protocols. In our study, after seeding two breast cancer cell lines in the 3D scaffold, we observed EMC deposition, cell adhesion to the stroma, and cell-to-cell junctions in a way that is similar to in vivo breast cancer conditions. We also showed tissue inhomogeneity due to the fibrous component and local differences in cell density.

Secondly, we reported good transfection efficiency obtained in a more realistic, and thus more predictable, therapeutic context for intratumoral plasmid DNA-based GET treatments to translate in vivo. Finally, we also reported very low cell mortality in this 3D model treated by GET, a critical aspect considering the translational potential of such a model.

In conclusion, the use of this new 3D model would make possible significant advances for drug delivery studies based on GET protocols, since it represents a predictable and efficient platform to evaluate electrotransfer conditions in a setting that reliably reproduces similar in vivo conditions.

## Supplementary Materials

The following are available online at https://www.mdpi.com/2072-6694/12/4/1043/s1, Figure S1: Representative images of electroporation procedure in 8-well chamber slides (A) using linear (B) or plate (C) electrodes, Table S1: GET efficiency after two GET protocols in 2D and 3D cell cultures, Table S2: Cell viability after two GET protocols in 2D and 3D cell cultures.
