# Supplementary material for: The Efficiency of Gene Electrotransfer in Breast-Cancer Cell Lines Cultured on a Novel Collagen-Free 3D Scaffold
Source: Cancers (Basel). 2020 Apr 23;12(4):1043. doi: 10.3390/cancers12041043 (PMC7226458; doi:10.3390/cancers12041043)
Supplement: Supplementary file 1 [file cancers-12-01043-s001.pdf]

Supplementary Material: The Efficiency of Gene Electrotransfer in Breast-Cancer Cell Lines Cultured on a Novel Collagen-Free 3D Scaffold

Elisabetta Sieni, Monica Dettin, Mariangela De Robertis, Bianca Bazzolo, Maria Teresa Conconi, Annj Zamuner, Ramona Marino, Flavio Keller, Luca Giovanni Campana and Emanuela Signori

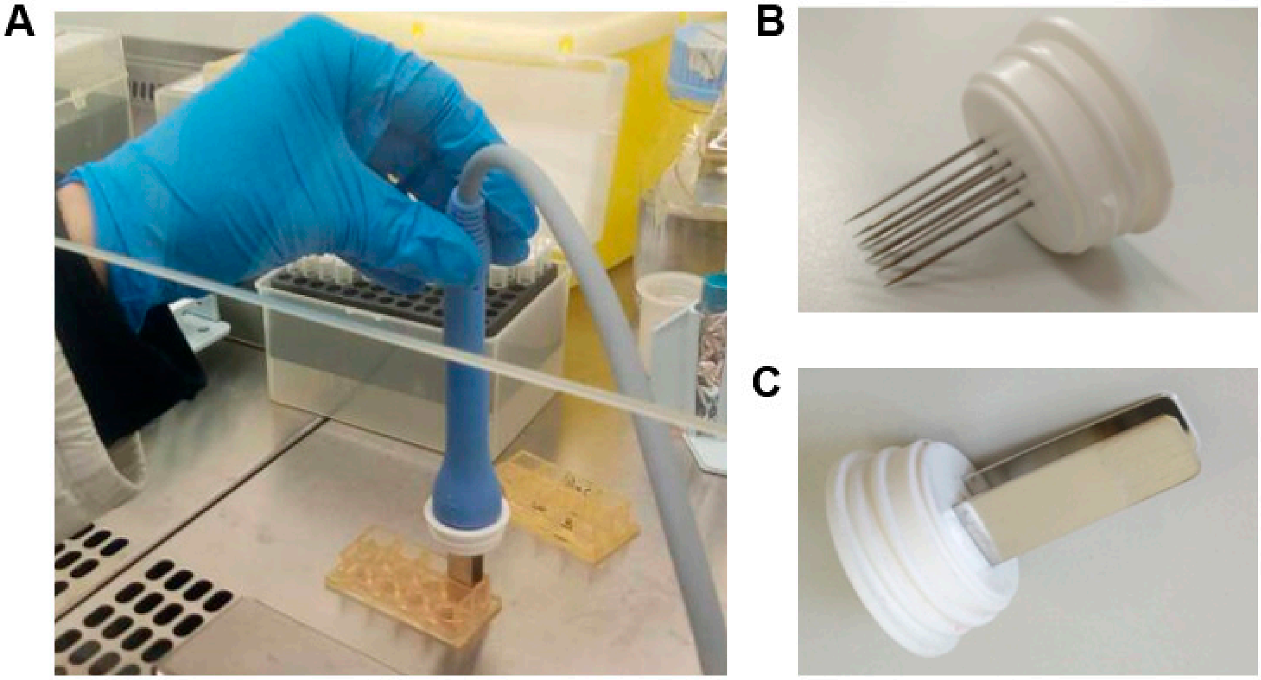

Figure S1. Representative images of electroporation procedure in 8-well chamber slides (**A**) using linear (**B**) or plate (**C**) electrodes.

Table S1. GET efficiency after two GET protocols in 2D and 3D cell cultures. Percentage of cell transfection of HCC1954 (**A**) and MDA-MB231 (**B**) cell lines in 2D and 3D cultures measured three days after EP (with a linear or plate electrode).

**A**	**HCC1954**		
	**2D**	**3D**	
GET protocol—Linear Electrode	20% (± 2,47)	12% (± 2,60)	$p < 0.01$
GET protocol—Plate Electrode	15% (± 2,16)	44% (± 12,48)	$p < 0.01$
	p = n.s.	$p < 0.0001$	
**B**	**MDA-MB231**		
	**2D**	**3D**	
GET protocol—Linear Electrode	13% (± 1,02)	11% (± 4,32)	p = n.s.
GET protocol—Plate Electrode	17% (± 2,12)	24% (± 2,16)	$p < 0.01$
	p = n.s.	$p < 0.01$	

Data are from two independent experiments and are displayed as Mean ± SD. Statistically significant differences are calculated using Student's *t*-test. Abbreviations: n.s, not significant.

Table S2. Cell viability after two GET protocols in 2D and 3D cell cultures. Viability of HCC1954 (**A**) and MDA-MB231 (**B**) 2D and 3D cell culture after three days post-GET using linear or plate electrodes. Percentage viability obtained using PrestoBlueTM Cell Viability assay. Control: untreated cells.

**A**	**HCC1954**		
	**2D**	**3D**	
GET protocol—Linear Electrode	81% (± 4,13)	81% (± 8,34)	p = n.s.
GET protocol—Plate Electrode	53% (± 1,28)	66% (± 2,30)	p = n.s.
	$p < 0.0001$	$p < 0.01$	
**B**	**MDA-MB231**		
	**2D**	**3D**	
GET protocol—Linear Electrode	65% (± 0,87)	86% (± 10,60)	$p < 0.01$
GET protocol—Plate Electrode	63% (± 3,51)	96% (± 5,24)	$p < 0.0001$
	p = n.s.	p = n.s.	

Data are from quadruplicate biological repeats and are displayed as Mean ± SD. Statistically significant differences are calculated using Student's *t*-test. Abbreviations: n.s, not significant.
